# An Enhanced Hidden Markov Map Matching Model for Floating Car Data

**DOI:** 10.3390/s18061758

**Published:** 2018-05-31

**Authors:** Mingliang Che, Yingli Wang, Chi Zhang, Xinliang Cao

**Affiliations:** School of Geographic Science, Nantong University, Nantong 226019, Jiangsu, China; dawnche@163.com (M.C.); benz1983@163.com (C.Z.); cxliang@mail.ustc.edu.cn (X.C.)

**Keywords:** satellite positioning systems, floating car data, map matching model, hidden Markov model, topological adjacency, traffic regulation

## Abstract

The map matching (MM) model plays an important role in revising the locations of floating car data (FCD) on a digital map. However, most existing MM models have multiple shortcomings, such as a low matching accuracy for complex roads, long running times, an inability to take full advantage of historical FCD information, and challenges in maintaining the topological adjacency and obeying traffic rules. To address these issues, an enhanced hidden Markov map matching (EHMM) model is proposed by adopting explicit topological expressions, using historical FCD information and introducing traffic rules. The EHMM model was validated against areal ground dataset at various sampling intervals and compared with the spatial and temporal matching model and the ordinary hidden Markov matching model. The empirical results reveal that the matching accuracy of the EHMM model is significantly higher than that of the reference models regarding real FCD trajectories at medium and high sampling rates. The running time of the EHMM model was notably shorter than those of the reference models. The matching results of the EHMM model retained topological adjacency and complied with traffic regulations better than the reference models.

## 1. Introduction

A vehicle’s location is a crucial geographic element in the performance of driver assistance, route navigation, vehicle monitoring and scheduling, and traffic management [1]. To obtain real-time vehicle location and auxiliary information, such as a time stamp, heading direction and speed, the usage of floating car technology (FCT) is becoming increasingly popular [2]. The information collected by FCT is known as floating car data (FCD). With FCD, a vehicle’s location can be mapped onto a digital map. However, FCD comprise raw material that is not precise due to measurement errors caused by the limited accuracy of the Global Positioning System (GPS) and the sampling error from sampling rates [3,4]. In addition, digital maps contain errors [5,6]; thus, the location of a vehicle shown on a digital map can be inaccurate. To solve this problem, map matching (MM) models have been rapidly developed over the past decade. At the same time, a large number of MM patent studies have been undertaken to meet the demands of car navigation manufacturers [7,8].

Most existing MM models can be characterized as either offline or online [9]. Offline models, which are also known as global models, batch process an entire input trajectory prior to generating a solution. These models attempt to obtain a curve that is as close as possible to a vehicle trajectory in a road network as the most likely road route. To evaluate the quality of the matching result, the Fréchet distance between the trajectory and the matched path in the road network is used [10,11,12].

One of the most widely used offline MM models is the spatial and temporal matching (STM) model [13]. The STM model uses spatial and temporal analyses to generate matching results. However, an important variable, specifically, the heading direction, is not considered in STM. In addition, the shortest path is computed using the A* algorithm [14]. In these cases, the matching accuracy is low, particularly the matching results that break this conditionality, i.e., the topological spatial relations among road segments. Chandio et al. [15,16] improved the STM model based on the locality of a road network to obtain the locality-based matching (LBM) model. The matching results indicate that the LBM model can reduce the total number of shortest path queries against the STM [15,16]. However, the improvement in the matching accuracy of the LBM model relative to STM is insignificant. Based on STM, Hsueh et al. [17] developed an MM model by introducing the directional analysis, namely, the spatio-temporal-directional based matching (STDM) model. The data validation shows that the STDM model outperforms the STM model and the other reference models. However, this model still does not consider traffic rules. When the vehicle and the road segment are in opposite directions, false matching frequently occurs.

Offline models tend to solve the MM of historical trajectories, whereas online models are appropriate for real-time FCD. Such models, which are also known as local or incremental models, use localizing matching strategies that partition an input trajectory into smaller segments and sequentially process them, which sometimes produces a suboptimal solution [9]. In [5], a comprehensive literature review of MM models that existed prior to 2006 was presented. In addition, the models were categorized into four groups: geometric models, topological models, probabilistic models and advanced techniques. As the demand for intelligent transport system (ITS) services has increased, MM models have been correspondingly developed. MM models integrate multiple types of techniques, and previously distinct categories have become ambiguous. The structure of MM models is becoming more complex, and the number of parameters is increasing rapidly. Based on key methods for location correction and frequency of use, MM models can be divided into five categories in this study: fuzzy logic models [18,19,20,21,22], hidden Markov models [9,23,24,25,26], weight models [1,20,27,28,29], topological models [26,28,29,30] and advanced models [2,31,32,33,34,35,36,37].

The fuzzy logic model applies to qualitative conditions, such as likeliness. Quddus et al. [21] developed an MM model that is based on fuzzy logic theory by considering factors such as the speed, heading direction, vehicle trajectory history, connectivity and orientation of the links and satellite geometric contribution to the positioning error. The matching results indicate that the fuzzy logic-based MM model provides a significant improvement over existing MM models in terms of identifying correct links and estimating the vehicle position on the links. However, the performance of the model was not validated for an urban road network due to the unavailability of high-accuracy GPS carrier-phase observations in the region. He et al. [20] proposed an improved fuzzy logic-based MM (FLMM) model and tested it on complex urban road networks. The model used a fuzzy inference system to address four state input variables that were described in the literature to estimate the behavior pattern weight of a taxi traveling on elevated roads. The test results indicate that the FLMM model outperforms the reference models and improves the MM accuracy, particularly for multi-layer roads. However, the fuzzy inference systems are very complex and computationally intensive [38]. Therefore, the FLMM model does not perform better than other models in terms of performance, which is very important in real-time map matching. In addition, the FLMM model was restricted to taxis and coupled the weight model, which is described below. Thus, the model inevitably involved numerous weight parameters that are constant and were not always efficient for all trajectory points.

The hidden Markov matching (HMM) model uses a hidden Markov chain to predict the road segment where a vehicle is located. For example, Newson et al. [23] presented an HMM-based model, called the Newson model, that explicitly accounts for measurement noise and the feasible routes in the road network. The study tested how the model responded when the location-sampling-rate decreased, and the measurement noise increased. The results revealed that the Newson model was not sensitive to certain types of noise and that the matching accuracy was barely degraded even with a sampling interval of 30 s. Subsequently, Goh et al. [9] developed an improved HMM model, called the Goh model, proposing two improvements over existing HMM models. The first improvement was the use of an optimal localizing strategy and the variable sliding window, which ensured online solution quality with uncertain future inputs. The second improvement was the novel combination of spatial, temporal and topological information using machine learning. Under a test of ground truth data, the Goh model was robust to noise and sparseness and viable for low-latency applications, such as traffic sensing. However, the time cost of the Goh model should be considered due to its use of the A* algorithm instead of topological spatial relations to calculate the distance discrepancy function. Challenges arise when adapting the parameters that are static and empirical in the Goh model to environmental settings such as urban or rural regions, where GPS accuracies may vary. The aforementioned models have a major weakness, i.e., the probability distribution of the next state depends only on the present state and not on the past or future states [39]. This results in the loss of contextual information when calculating the probability distribution of the next state. Thus, Jagadeesh et al. [39] proposed a novel HMM model, the HMM-RCM model, by considering the concept of drivers’ route choice. The assessment results show that the HMM-RCM model outperforms the reference models. Nevertheless, the performance of HMM-RCM is not validated in the practical deployment scenario. Moreover, the HMM-RCM model does not take advantage of heading or direction data and does not consider the traffic conditions too.

The weight model uses the weight coefficients to minimize the total MM error in terms of identification of the correct links. For example, Velaga et al. [27] described an enhanced weight-based MM (EWMM) model in which the weights were determined from real-world field data using an optimization method. They introduced two new weights for turn restrictions at the junctions and link connectivity. The matching results revealed that the EWMM model was superior to reference models, particularly at junctions. However, the EWMM model contains numerous parameters and coefficients that are static and restricted to a specific operational environment. Developing some methods to calculate the weights for each GPS point based on its special circumstances may improve the output [38].

The topological model uses the geometry of the links and the connectivity and contiguity of the links to solve the MM problem. For example, Ren et al. [26] considered the spatial connectivity as the input variable of the natural exponential function to calculate the transmission probability between two road segments. In topological models, the topological information generally couples with other techniques, such as fuzzy logic theory [20], HMM [26], weight [27] and Dempster–Shafer (D–S) theory [30]. For instance, Quddus et al. [28] developed a model that included a topological technique and a weight technique. The expression of topological information is typically explicit, i.e., quantitative to the formula or implicit, i.e., implemented using shortest-path algorithms. The model in [26] uses an explicit topological expression, whereas the model in [28] uses an implicit topological expression. The running time of the former was found to be shorter than that of the latter [15,16].

The advanced models contain the Kalman filter model [31,32], the particle filters model [33,34], the generic model [35], the conditional random field model [36], and the probabilistic model [2,37]. In these models, the structures are novel. They may have advantages in terms of correct road identification and matching accuracy [38], e.g., the correct link rate of the generic model being up to 99.1%, just lower than that of the Newson model. The computational costs of these models are usually high [35,36], e.g., the calculation of Kalman Gain in the Kalman filter models referring to a large number of iterations. Thus, these models are likely to take a long time to run. In this case, they may not be suitable for real-time applications [35]. To overcome this shortcoming, some studies have optimized the implemented technologies, e.g., adopting high performance computing (HPC). Wang et al. [32] used the parallelized computation of HPC in the MM model. The experimental results show that the matching efficiency is 25% higher than that of the same number of stand-alone computers. Huang et al. [40] deployed the MM model into the cloud computing environment of HPC. The test results show that this approach has an obvious efficiency advantage on massive vehicle tracking data processing. Although the running efficiency is improving, the model structure becomes very complex. Some studies have indicated that the advanced models are inferior in terms of implementation simplicity and performance [38].

Some findings may be extracted from the above analysis: (1) not all MM models pay sufficient attention to the directionality of roads and traffic rules; (2) not all models accommodate various types of floating cars; (3) the fuzzy logic models and the advanced models are not dominant in terms of implementation simplicity and performance; (4) the weight models balance simplicity and accuracy, but contain numerous empirical parameters and coefficients [38]; (5) the HMM models are implemented simply and adopted widely but cannot take advantage of historical and future FCD information [17]; (6) the topological expression in most topological models is implicit and implemented by the shortest-path computation, whose running time is quite heavy [41]. Although existing MM models are never perfect and cannot satisfy the requirements of all ITS applications and services [5], the weaknesses of MM models imply that additional improvements of MM models are essential and impending. In this study, to reduce the computational cost and the number of parameters and increase the matching accuracy of complex urban road networks, particularly multi-layer roads and parallel roads, we propose an enhanced MM model based on HMM. The proposed model—which adopts explicit topological expressions, uses historical FCD information and introduces traffic rules—is designed for all types of floating cars. Using ground truth data, we test the performance of the proposed model against the reference MM models.

## 2. Materials and Methods

### 2.1. Dataset Description 

**Figure 1:**
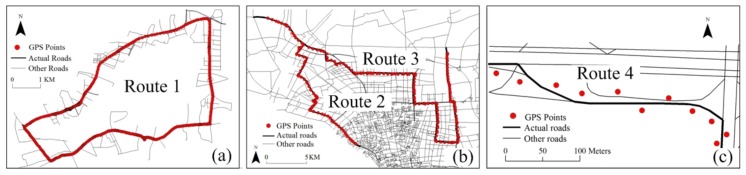
Four actual routes on three datasets (**a**–**c**).

The trajectory data and the corresponding road networks contain three datasets. In the previous study, the first dataset (dataset 1) was collected in Guangzhou using a handheld GPS device with a sampling interval of 5 s (the other intervals can be acquired by sampling) [20]. In dataset 1, there are 421 roads from a road network consisting of ordinary roads, elevated roads and the roads below these roads. The actual route, termed route 1 (Figure 1a), is divided into two groups: simple roads, also known as ordinary roads, with simple intersections that do not have parallel roads in the buffer of 50 m, and complex roads including multi-layer roads and parallel roads, e.g., an elevated road and its side roads and complex intersections. The route covers 11 different roads and 72 links for a total length of 20,258 m. To effectively evaluate the matching models, this route was sampled three times with 482, 671 and 566 sampling points.

The second dataset (dataset 2) was collected from the actual trajectories of freight cars in Nantong. In dataset 2, the road network stems from the vectorization of the remote sensing images and involves 14,792 roads that traverse the entire city. The actual routes, termed route 2 and route 3, are shown in Figure 1b. Both routes contain simple roads and complex roads. Route 2 includes 49 links with a total length of 30,564 m. Route 3 comprises 92 links with a total length of 45,530 m. On both routes, the sampling interval of each floating carwas30 s.

The third dataset (dataset 3) was collected from the actual trajectories of cars in Xian. In dataset 3, the road network is vectorized as described in [30]. The GPS points of the floating cars are also vectorized as described in [30]. The actual route, i.e., route 4, and the corresponding GPS points are shown in Figure 1c. On this route, the sampling interval was 30 s.

In the trajectory data, the necessary variables include the identifying number (ID), time, longitude, latitude and heading direction. Missing values of longitude or latitude were estimated using linear interpolation. Missing and abnormal values of the heading direction were not addressed to ensure that the robustness of the matching model could be verified. For describing conveniently and clearly in the following context, the trajectory data with interval ranges from 5 s to 150 s was categorized into three groups, i.e., the low, moderate and high sampling rate FCD. Similar to previous studies [13,17,42,43], the interval of low sampling rate FCD is set to exceed 60 s, the interval of high sampling rate FCD is shorter than 40 s, and the moderate frequency FCD is in between. In the road network, the required variables were the road ID and direction. The road direction of the vector road network was auto-calculated using the “Linear Directional Mean” tool of current Geographic Information System (GIS) software, i.e., ArcGIS. In one-way roads, the road direction was examined and aligned manually.

### 2.2. Model Schema

#### 2.2.1. Definitions

• Definition 1 (observation point)

The instantaneous status of a vehicle is considered the observation point, i.e., the GPS point in the study, which has both location (longitude and latitude) and attributions (e.g., a timestamp, heading direction, and velocity). The sequence of the observed points forms a GPS trajectory.

• Definition 2 (candidate feature, CF)

**Figure 2:**
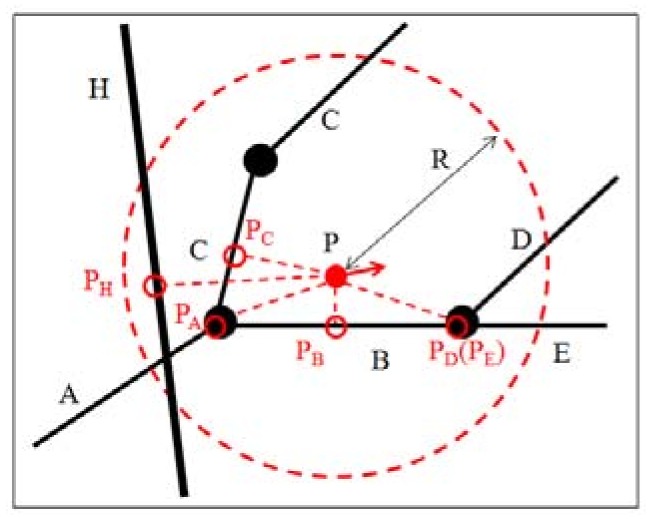
CFs and their spatial relations. The CFs A, B, C, D, and E are urban ordinary roads, and the CFH is an urban elevated road. The point P is the observation point, whose shortest distances to various CFs are denoted by red dashed lines. The candidate points are denoted by red hollow circles. The intersection points among CFs are denoted by black solid circles. The candidate region is denoted by the red dashed circle. The letter R denotes the candidate radius.

The feature is important in a GIS and is abstracted as a representation of a real-world object on a map. The feature contains both geometry and attribution. In this study, the geometry type refers to the line only, i.e., the road segment that contains at least two sequential vertices and at least one corresponding edge. As shown in Figure 2, the road segments A, B, C, D, E and H are features. Note that feature C has multiple vertexes and multiple edges. To match the observation point to the correct road segment, every feature has the potential to be a candidate. However, most features are not suitable as candidates because they are located too far from the observation point or because their heading directions are significantly inconsistent with the observation’s. Thus, to improve the matching efficiency, the creation of a buffer that uses the observation point P as the center and the given distance R as the radius is essential. These features, which are located in the buffer, are referred to as CFs, which have an additional chance of becoming matching correct road segments. The spatial relations among different CFs contain only two types of relations: adjacency and separation. In Figure 2, the features A, B and C are adjacent. Features A and D are disjointed, as are features A and H. Feature H is an urban elevated road, and feature A is an ordinary road. Their intersections are empty.

• Definition 3 (candidate point, CP)

The point in the CF that is closest to the observation points is defined as a CP. Every CF has only one CP. If the projection of the observation point onto the CF is located between its endpoints, then choose the geometry projection as the CP; otherwise, choose the end point that is closer to the observation point with regard to the Euclidean distance, as shown in Figure 2. The projection P_B_ is the CP of P in feature B, and the endpoint P_D_ is the CP of P in feature D. The CFs D and E have the same CP. Among the CPs, the point that maximally satisfies the matching rules is considered a confident point and added to the resulting list. The corresponding CF is considered a confident feature and added to the results list. The matching rules are presented in the following context.

#### 2.2.2. Observation Probability

Similar to previous studies [13,23,26], the observation probability model refers only to the current status, i.e., the current CF and the current observation point. This status is controlled by the distance and azimuth.

• Distance constraint probability

The distance constraint probability is computed as:

(1) $$P_{dis}=exp(-\beta_{dis}\cdot dis)$$

(2) $$dis=acos(sin\varphi_{obs}\cdot sin\varphi_{candi}+cos\varphi_{obs}\cdot cos\varphi_{candi}\cdot cos(\lambda_{obs}-\lambda_{candi}))\cdot R$$

where *P_dis_* is the distance constraint probability calculated by the exponential function, and $\beta_{dis}$ is the adjustment coefficient. The argument *dis* is the spherical distance between the observation point and the candidate point, which is calculated based on the spherical law of cosines. The parameters φ and λ denote the latitude and the longitude, respectively. *R* represents the Earth’s radius.

• Azimuth constraint probability

**Figure 3:**
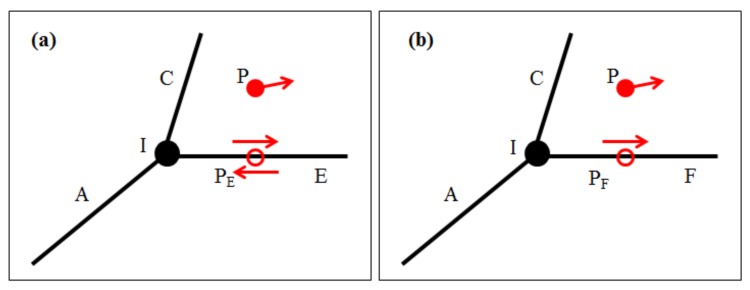
Comparison of azimuths between the observation points and the candidate points. The observation points are denoted by red solid circles. The candidate points are denoted by red hollow circles. The azimuths of the points are denoted by red solid arrows. The intersection among the CFs are denoted by black solid circles. The road segments E and F comprise a two-way road (**a**) and a one-way road (**b**), respectively.

The azimuth constraint probability is defined as:

(3) $$P_{azi}=|cos(\alpha_{obs}-\alpha_{candi})|$$

where *P_azi_* is the azimuth constraint probability, which is quantified by the absolute cosine function. The argument $\alpha_{obs}$ is the instantaneous heading direction of the observation point, and $\alpha_{candi}$ denotes the azimuth of the candidate point. As shown in Figure 3, the candidate points *P_E_* and *P_F_* have two directions and one direction, respectively. Thus, Equation (3) can be used to evaluate the included angle between the direction of the observation point and the CF.

• Traffic rule constraint probability

The traffic rule constraint probability is defined as:

(4) $$P_{traf}=\{\begin{array}{cc} \delta_{dir}\leq \delta_{0} & 1\\ \delta_{dir}>\delta_{0} & 0 \end{array}$$

(5) $$\delta_{dir}=\left|min(\right|\alpha_{dir1}-\alpha_{obs}\left|,\right|\alpha_{dir2}-\alpha_{obs}\left|)-180\right|$$

where *P_traf_* is the traffic rule constraint probability, whose value is equal to one or zero, corresponding to the traffic rules, e.g., driving against traffic on a one-way road is never allowed. $\delta_{0}$ is a threshold with the unit of degree. $\delta_{dir}$ is calculated by Equation (5), which is related to the heading direction of the observation point and the directions of the CFs, i.e., $\alpha_{dir1}$ and $\alpha_{dir2}$. Generally, roads can be divided into two-way roads and one-way roads. As shown in Figure 3, the heading direction of the observation point is consistent with the CFs, that is, the traffic rule of the vehicle is satisfied.

• Observation probability

Combining Equations (1), (3) and (4), the observation probability is defined as:

(6) $$P_{obs}=P_{dis}\cdot P_{azi}\cdot P_{traf}$$

where *P_obs_* is the observation probability, which comprehensively considers the constraint conditions, including the distance, the azimuth and the traffic regulation between the observation point and the CF.

#### 2.2.3. Transmission Probability

• Topology constraint probability

The topology constraint probability, as described in the previous study [26], is computed as:

(7) $$P_{topo}=exp(-r)$$

(8) $$r=\{\begin{array}{ccc} C & & fea_{1}\neq fea_{2},fea_{1}\cap fea_{2}\neq \emptyset \\ 0 & & fea_{1}=fea_{2},fea_{2}\cap point_{proj}\neq \emptyset \\ \infty & & \mathrm{other} \end{array}$$

where *P_topo_* is the topology constraint probability, and *r* is the topology stamp. The *r* value involves the spatial relation between two CFs. If two CFs are unequal and their intersection is not empty (i.e., if the two features are adjacent), then *r* is a positive constant. If two CFs are overlaid and the intersection between one feature and the projective point of the observed is not empty (i.e., if the two features are equal and if one feature contains the projective point of the observed), then *r* is zero. In other cases, *r* is infinite. Equation (7) indicates that larger value of the topology constraint probability corresponds to a greater chance of transmission from the previous CF to the current CF.

• Aspect constraint probability

The aspect constraint probability is computed as:

(9) $$P_{asp}=exp(-\beta_{asp}\cdot |\alpha_{obs}-\alpha_{candi}^{\prime }|)$$

where *P_asp_* is the aspect constraint probability, $\beta_{asp}$ is the adjust coefficient, $\alpha_{obs}$ is the heading direction of the observation point, and $\alpha_{candi}^{\prime }$ is the azimuth of the CF relative to the crossover point with the previous CF. Equation (9) indicates that the possibility of transmission from the previous CF to the current CF increases if the azimuth of the observation point more accurately approximates that of the current CF.

• Shape constraint probability

(10) $$P_{shp}=exp(-\beta_{shp}\cdot \sqrt{\sum_{i=1}^{m}\alpha_{i}^{2}})$$

(11) $$\alpha_{i}=acos(\frac{\vec{CP_{i}CP_{i+1}}\cdot \vec{OP_{i}OP_{i+1}}}{\left|\vec{CP_{i}CP_{i+1}}|\cdot |\vec{OP_{i}OP_{i+1}}\right|})$$

where *P_shp_* is the shape constraint probability, $\beta_{shp}$ is the adjusted coefficient, and $\alpha_{i}$ denotes the vector included angle. The vector $\vec{CP_{i}CP_{i+1}}$ denotes the directed line segment from the *i*th candidate point to the (*i* + 1)th candidate point. Similarly, the vector $\vec{OP_{i}OP_{i+1}}$ denotes the directed line segment from the *i*th observation point to the (*i* + 1)th observation point. Equation (10) demonstrates that a greater similarity of the trajectory formed by the observation points to the previous and current CFs corresponds to a higher chance of transmission from the previous CF to the current CF.

• Transmission probability

Combining Equations (7), (9) and (10), the transmission probability is defined as:

(12) $$P_{trans}=P_{topo}\cdot P_{asp}\cdot P_{shp}$$

where *P_trans_* is the transmission probability, which can ensure the logical rationality of the feature transmission. However, the transmission probability is more suited for high sampling rates. For compatibility with low sampling rates, a shortest path algorithm is used to amend the transmission probability and is defined as:

(13) $$P_{trans}=exp(-\beta_{sp}\cdot |\frac{dis(CP_{i-1}\to CP_{i})}{dis(OP_{i-1}\to OP_{i})}-1|)$$

where $\beta_{sp}$ is the adjusted coefficient, $dis(CP_{i-1}\to CP_{i})$ is the length of the shortest path calculated using Dijkstra’s algorithm from the candidate point *CP_i_*_−1_ to the candidate point *CP_i_*, and $dis(OP_{i-1}\to OP_{i})$ is the spherical distance between the observation point *OP_i_*_−1_ to the observation point *OP_i_*.

#### 2.2.4. Matching Solution

Based on the observation probability and the transmission probability, the hidden Markov model is applied to solve the MM problem. The output probability is formed as:

(14) $$P(X_{n+1}|X_{n})=(P_{trans}^{n+1}\cdot P_{obs}^{n+1})\cdot P(X_{n}|X_{n-1})$$

where *P* is the output probability. The subscript *n* represents the sequence number of observation points. To obtain the most likely sequence of hidden states, i.e., the best path, the Viterbi algorithm [44] is employed.

**Figure 4:**
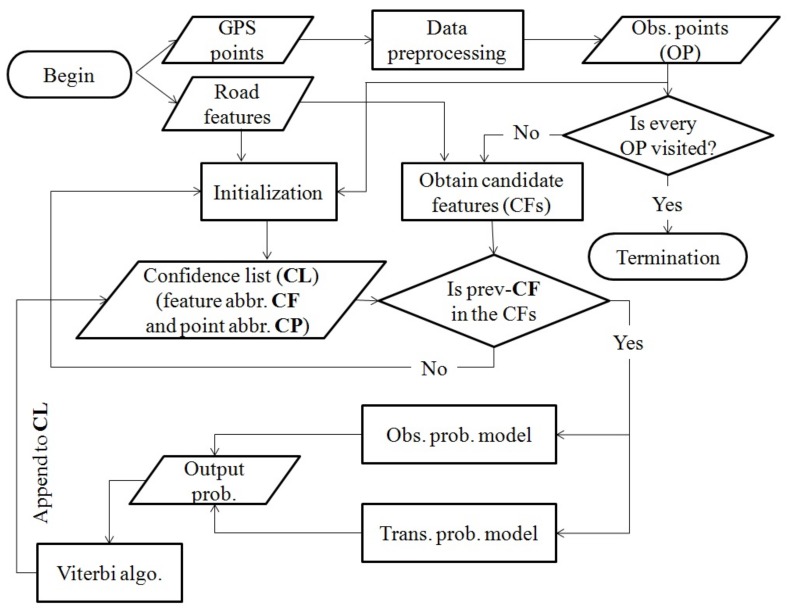
Flow chart of the proposed model.

The flow chart of the entire model is shown in Figure 4. The life cycle primarily involves the beginning, data preprocessing, initialization, identification of CFs, calculation of matching probabilities, and termination. Data preprocessing refers to sorting the GPS points in ascending order by GPS time stamp (necessary for offline data), repairing the missing values of location data and rejecting useless GPS points. Initialization is employed to calculate the best matching feature of the first observation point. This step relies on the observation probability model and returns two confidence lists (**CL**s). The first **CL** is the confidence feature list, and the second **CL** is the confidence point list. The process of identifying CFs generates a set of CFs that are based on the candidate radius. The set reserves the geometry topologies and attributes of the original features. Then, the process checks whether the last feature in the **CL**s is in the set of CFs. If so, then the process calculates the matching probabilities; otherwise, the process shifts to the initialization step for correcting the matching errors. With the observation probability model and the transmission probability model, the output probability is calculated. Based on the Viterbi algorithm, the confidence feature and confidence point are appended to the **CL**s. After every observation point is visited, the algorithm terminates.

### 2.3. Reference Models

The performance of the proposed model, which is termed an enhanced hidden Markov map matching (EHMM) model, is compared with that of two popular models.

The first popular model is the ordinary HMM model. In HMM, the observation probability and the transmission probability are separately calculated [23]. The observation probability is concerned only with the distance between the observation point and the corresponding candidate point. The transmission probability is related to the difference between the distance between two adjacent observation points and the distance between two corresponding candidate points. The smaller the absolute value of the distance difference, the greater the likelihood of transmission from the previous feature to the current.

The second popular model is the STM model, which includes two modules, namely, the spatial analysis and the temporal analysis, as discussed in the literature [13]. The spatial analysis incorporates the observation probability and the transmission probability. Similar to HMM, the observation probability and the transmission probability are related to the distance. However, the probability expressions of STM differ significantly from those of HMM. The temporal analysis refers the average velocity. In the present study, obtaining the velocity of the road segment was prohibitively challenging; thus, the temporal analysis was abandoned. For this reason, the STM model is referred to as the SM model hereinafter.

Moreover, the EHMM model was tested against newer and more efficient models, i.e., FLMM and ATMM. The FLMM model is based on fuzzy logic and weights and uses two matching methods: point-to-curve matching and point sequence matching, which are described in [20]. The ATMM is an advanced topological MM model that uses D–S theory in the meantime to improve the application to a high-density road network. A detailed description of ATMM is in [30]. To compare the FLMM and ATMM model matching results, the input data for EHMM are the dataset 1 used in [20] and the visual matching result with trajectory points in [30]. In this case, the comparison is performed between the results of EHMM and the results of the two models published in the literatures.

### 2.4. Evaluation and Analysis Approaches

The approach used to evaluate the matching accuracy among the three models is defined as:

(15) $$AP=\frac{M_{pt}}{N_{all}}$$

where *AP* is the point-matching accuracy, *M_pt_* denotes the number of observation points matched to the correct features, and *N_all_* is the total number of observation points.

To analyze variations in the matching accuracy and the running time among the three models at different sampling intervals, a simple linear regression model was employed.

In addition, the following assumptions are made to compare the running times of the three models. (1) All models are running on the same hardware device, i.e., a personal computer (PC) equipped with a 4-core CPU and a 4-GB memory-chip. (2) All models are running on the same operating system (OS) in PC, i.e., 32-bit Windows 7 OS. (3) All models are designed as single-thread programs and implemented in Java.

## 3. Results Analysis

### 3.1. Evaluation of Matching Accuracy at a High Sampling Rate 

**Figure 5:**
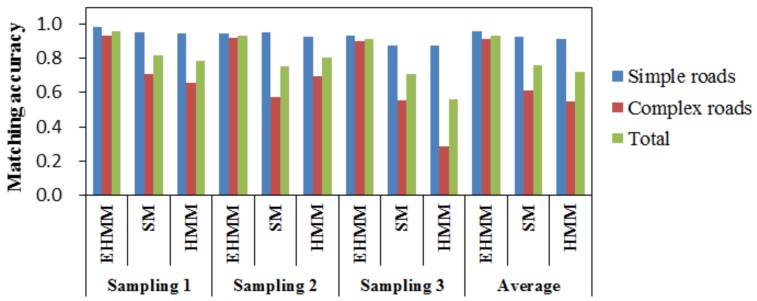
Comparison of matching accuracy among the various models on route 1. The last three columns list the mean of the three samplings in dataset 1.

Dataset 1 was used to verify the matching accuracy of the three models. As shown in Figure 5, the matching accuracy of these models in three samplings can be compared. For the total roads, the matching accuracy of EHMM was significantly higher than that of SM and HMM. In the three samplings, the AP mean of EHMM was 0.96. The results of models SM and HMM were similar in the previous two samplings. In the last sampling, the performance of SM was moderate, and the performance of HMM was poor.

**Figure 6:**
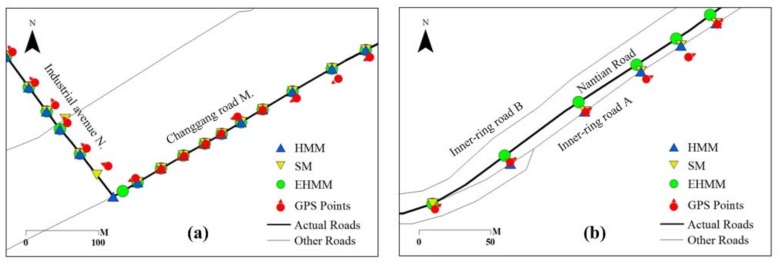
Comparison of matching performance among the various models on simple roads (**a**) and complex roads (**b**).

For simple roads, the three models generally performed well. The AP of EHMM was only 3.4% higher than that of SM and HMM. The corresponding results are shown in Figure 6a. Three models corrected the GPS points to the real roads well. For complex roads, the difference in the matching results among the three models was large. Among the three samplings, the matching results of EHMM were satisfactory. The minimal AP of EHMM was 0.9. The next model was the SM model with an AP mean of 0.61. The matching results of the HMM model were satisfactory between the previous two samplings but were unsatisfactory in the last sampling, with a matching accuracy less than 0.55. The matching results among the three models are shown in Figure 6b. Two roads lay parallel to the Nantian road. The positions of the GPS points were seemingly close to the inner-ring road A. The SM and HMM models mismatched many points to this road. However, the EHMM model performed well. According to the statistics of AP for simple roads and complex roads, the results indicate that the performance of the three models for simple roads was better than that of the three models for complex roads.

### 3.2. Comparison of Matching Accuracy at Various Sampling Rates

**Figure 7:**
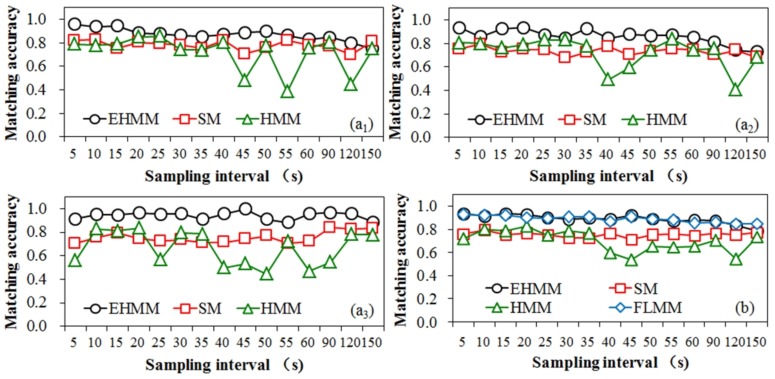
Comparison of matching accuracy at various sampling intervals based on dataset 1. In (**a**), the subscript numbers 1, 2 and 3 denote the first, second and third samplings, respectively, in dataset 1. (**b**) represents the average matching accuracies among the three samplings.

Figure 7 compares the matching quality of the three models at different sampling intervals based on dataset 1. For the three samplings, the matching quality of EHMM and SM was stable, with an average standard deviation of 0.06 and 0.04, respectively. For HMM, the matching results exhibited a large fluctuation with an average stand deviation of 0.14. With various sampling intervals, the relative positions between the GPS points and the roads exhibited randomness. Thus, the spatial positions of the GPS points had a significant impact on the matching results of HMM but a minimal impact on EHMM and SM. 

**Figure 8:**
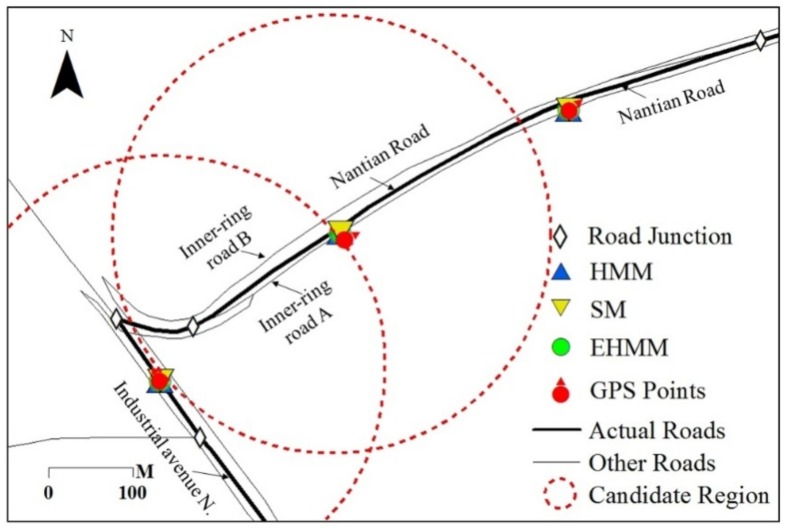
Weakening of the topological constraint with an increase in the sampling interval.

As the sampling interval increases, the matching accuracy of three models presented declining trends at various levels. The matching accuracy of EHMM significantly declined (*slope* = −0.74 × 10^−2^, *R*^2^ = 0.71) with increasing sampling intervals. The SM model also presented a downward but not prominent trend (*slope* = −0.12 × 10^−2^, *R*^2^ = 0.24 × 10^−2^). For the HMM model, the evidence of the downward trend consisted of a slope of −0.12 × 10^−1^ and an *R*^2^ value of 0.33. This finding reveals that the length of sampling interval primarily affected the EHMM model. As depicted in Figure 8, the GPS point was mismatched when the vehicle drove from the industrial avenue N to the inner-ring road A. Two junctions existed between the two roads. The topological relation between the feature in which the vehicle was projected to the industrial avenue N and the feature in which the vehicle was projected to the inner-ring road A was separated by a divergent road segment. As the sampling interval increased, the topological constraint weakened. The SM and HMM models resisted the topological constraint; the length of the sampling interval minimally affected them.

Although the matching accuracy of the three models decreased with increasing sampling intervals, the AP of the EHMM model was higher than that of other models at most sampling intervals, particularly at high sampling rates. For the SM and HMM models, the comparison became challenging. Prior to the sampling interval of 35 s, the AP of HMM was slightly higher than that of the SM model. Subsequently, in contrast, the SM model was predominant. When the sampling rate decreased, the shortage of topological constraints of the EHMM model tended to result in the shortest path, which was also adopted by the SM model. Thus, the similarity among the matching results between the EHMM model and the SM model gradually increased with increasing sampling intervals.

Figure 7b also shows a comparison of the matching performances between EHMM and another more efficient MM model, i.e., FLMM, based on dataset 1. It can be seen that the overall matching performances of the two models are nearly identical. The statistics show that the matching accuracy of EHMM, with a mean AP of 0.91, is higher than that of FLMM, with a mean AP of 0.90, at high sampling rates. The mean AP of EHMM is also higher than that of FLMM at moderate sampling rates, by 1%. At low sampling rates, the matching accuracy of EHMM (*AP_mean_* = 0.84) is inferior to that of FLMM (*AP_mean_* = 0.85), but only by 1%. As the sampling interval increases, the matching accuracy of the FLMM model exhibits a significant declining trend (*slope* = −0.56 × 10^−2^, *R*^2^ = 0.85). This indicates that the length of the sampling interval also significantly affects the FLMM model.

### 3.3. Analysis of the Running Time

**Figure 9:**
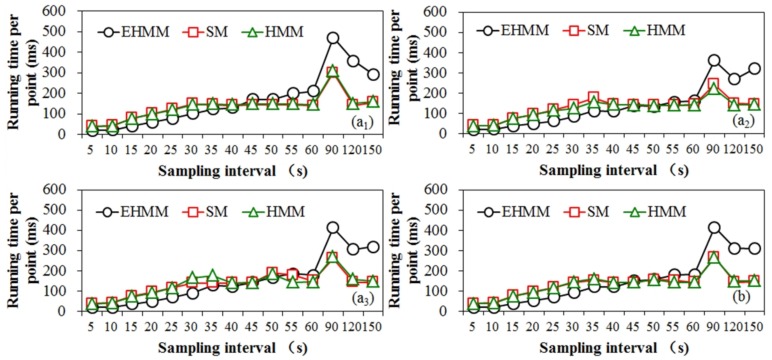
Comparison of running times for various sampling intervals based on dataset 1. In (**a**), subscript numbers 1, 2 and 3 represent the first sampling, second sampling and third sampling, respectively, in dataset 1. (**b**) represents the average running time among the three samplings.

**Figure 10:**
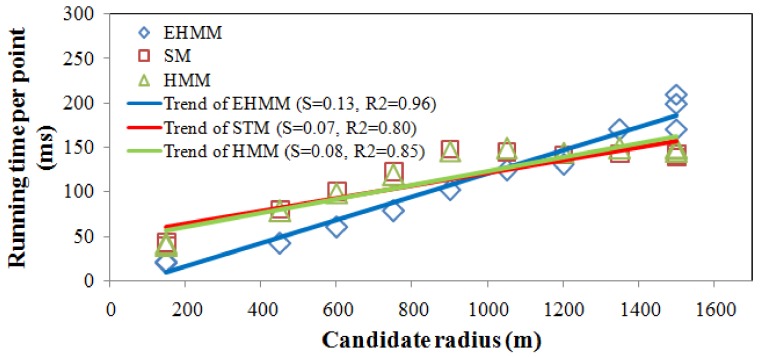
Influence of the candidate radius on running time.

A comparison of running times for various sampling intervals on route 1 is shown in Figure 9. The running times among the three models increased as the sampling interval increased. The running time of the SM model was similar to that of the HMM model. The difference in the running time between the EHMM model and the other models is notable. Prior to the sampling interval of 40s, the running time of the EHMM model was lower than that of the other models. Over this period, the EHMM model calculated that the transmission probability primarily depended on the topological constraint, which worked well, particularly for the high sampling rate. The SM and HMM models adopted the shortest path to calculate the transmission probability. In this study, the candidate radius of the models was dynamic and related to the sampling interval. As shown in Figure 10, the running time was significantly positively correlated with the candidate radius for the three models. When the sampling rate was high, calculating the topological relation was much faster than computing the shortest path based on the uniform candidate radius.

Subsequently, EHMM requires increasingly longer amounts of time. For sampling intervals exceeding 90 s, the difference in the running time between the EHMM model and the other models was significant and averaged 158 ms. As previously mentioned, when the sampling rate decreased, the topological constraint weakened. The EHMM model used the shortest path to correct the transmission probability. The running time of the EHMM model included the above two scenes. Thus, the running time of the EHMM model was slower than that of the other models.

The maximum running time, i.e., the time required for the EHMM model to process one GPS point, was approximately 7 s, which was substantially shorter than the sampling interval. Thus, the EHMM model is also appropriate for the operation.

### 3.4. Results on Real Data

**Figure 11:**
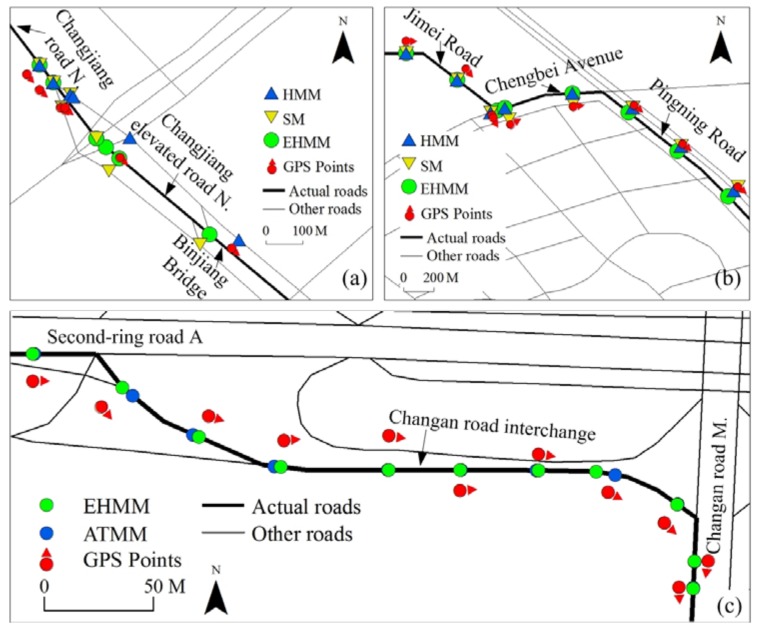
Comparison of matching performances among MM models for part of route 2 (**a**), part of route 3 (**b**) and route 4 (**c**).

A comparison of matching performance among three models based on dataset 2 is shown in Table 1. Figure 11 shows a visual comparison of the three models for parts of two roads. The matching accuracy of three models was generally high on route 2, which includes both simple roads and complex roads. However, the EHMM model was better than the other two models. The AP of the former was close to 0.9, whereas that of the latter was lower than 0.85. The difference between them was expounded in the complex roads. As shown in Figure 11a, after the vehicle drove into Changjiang elevated road N, which was a complex road, the matching results of the HMM and SM models were poor. The SM model violated the topological adjacency between the two roads. The HMM model violated the traffic regulations. On this road, the sampling interval was 30 s. The running time of EHMM was slightly shorter than that of the other models. On route 3, the matching performance of the EHMM model was superior to that of the other two models with regard to matching accuracy and running time. A visual comparison among the three models is shown in Figure 11b.

The EHMM model was also tested against another newer model, i.e., ATMM, based on dataset 3. Obviously, the matching results for the two models that are presented in Figure 11c are all satisfactory. They all match the GPS points to the actual roads, although their post-matching points are not always coincident. On this road, the sampling interval was 30 s. The running time per point for EHMM was 247 ms. However, the running time of the ATMM model is unknown because the computational cost of the ATMM model was not reported in [30]. Thus, no comparison of the running times between the two models is presented.

## 4. Discussion

The results of EHMM demonstrate that matching produces superior performance during the verification of real trajectory data relative to the reference models. However, on complex roads, such as multi-layer roads and parallel roads, the challenge of accurate matching is enormous because of the quality of the FCD, the model structure and the corresponding parameters.

**Figure 12:**
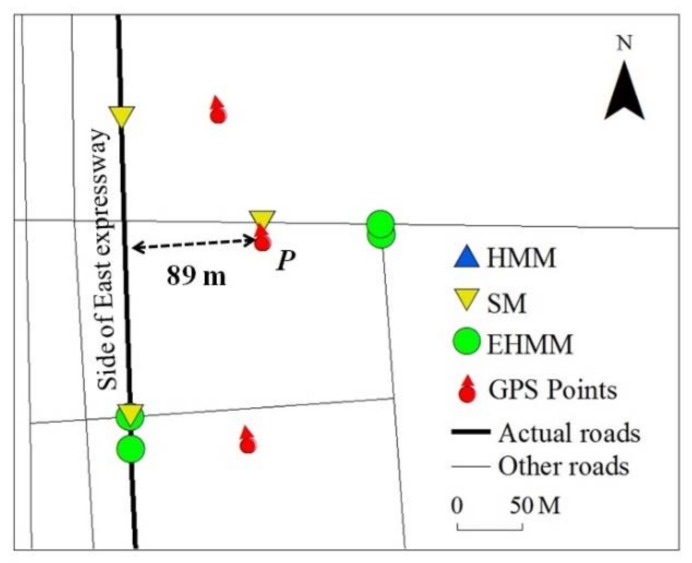
Challenges of MM for models on parallel roads.

**Figure 13:**
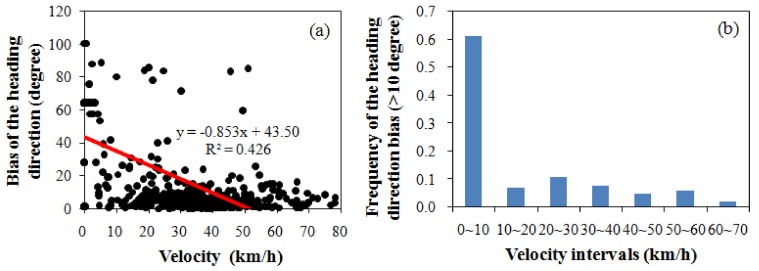
Influence of velocity on heading direction. (**a**) represents the correlation between the velocity and the heading direction bias. (**b**) displays the frequency histogram of the heading direction bias, which exceeds 10 degrees at various velocity intervals.

The quality of the FCD is the fundamental force for improving the matching accuracy of the model, particularly for the intersection of an elevated road and a side road. In the FCD, the location and the heading direction are important. Many uncertainties exist that arise from GPS measurement error, the quality of the terminal device and the influence of the high-street density in urban areas. When the bias of the location is minor, the vehicle position can be effectively corrected. When the bias is significant (e.g., in Figure 12, in which the location bias is 89m), the map matching of the models becomes challenging. The uncertainty of the heading direction causes additional challenges, particularly for low velocities. A vehicle typically decelerates when it encounters traffic jams or road turn-offs. A slower velocity increases the uncertainty in the heading direction. Figure 13 shows the influence of velocity on the heading direction. A significant passive correlation (*slope* = −0.853, *R*^2^ = 0.426) between the velocity and the heading direction bias can be observed. The bias of the heading direction increases by 35 degrees for each 10 km/h reduction in velocity. Figure 13b displays the frequency histogram of the heading direction bias, which exceeds 10 degrees at various velocity intervals. When the velocity is less than 10 km/h, the accuracy of the heading direction is extremely unsatisfactory. When the velocity is less than 7.2 km/h, the majority of the heading directions contain noise, which increases the MM challenges when the model determines that a vehicle is driving on an entrance (exit) ramp of the elevated road or the side road.

When the quality of the FCD is stable, the model structure and parameters compose the crucial solution to match the vehicle locations to the correct roads. In the structure of EHMM, elements such as the shortest distance, heading direction, traffic regulation, topology and shape similarity are comprehensively considered. EHMM adopts an explicit equation to express the topological spatial relation between two features. The depth of topological relation is one, i.e., two interconnected features need an adjacent relation, which is effective for high and moderate sampling rates. For the lower sampling rate, the topological relation is weakened (Figure 8). In the complex road network, two detached road features may remain connected if they contain other adjacent features; however, their topological relation is separated. This finding explains why EHMM employs the shortest path to remedy the shortage of the topological constraint at the lower sampling rate. However, the constraint equation based on the shortest path presents a challenge for the running time and matching accuracy. Beyond the topology, the structure in terms of other elements needs additional research. In addition, the velocity variable and the data assimilation method should be addressed using EHMM.

In EHMM, the parameters were set to the empirical constants. Due to the quality of the FCD and the vector map, the parameters were not always sufficient. Thus, optimization of the model parameters warrants future research.

The MM performance of EHMM was evaluated through a comparison with the foundational reference models. For a comprehensive evaluation of EHMM’s performance, this model was also tested against newer and more efficient models, i.e., FLMM and ATMM. As seen from the results analyzed above, the EHMM model is comparable to these two models in terms of matching accuracy. However, no comparison of the running time of EHMM with those of the two models was performed because the computational costs of these two models are not reported in [20,30]. In addition to the FLMM and ATMM models, there are many other excellent MM models. However, it is very difficult to compare them with EHMM. On the one hand, the trajectory data and the road network data used in these models are difficult to obtain. On the other hand, the coding implementation of these models is troublesome and difficult. The literature presents the theory of these models but does not detail model implementations. During model implementation, certain methods and parameters are fraught with uncertainty. Accordingly, difficulty arises in implementation efforts. Therefore, in future research, cooperation with other authors who have designed MM models must be established for the further improvement and evaluation of EHMM.

## 5. Conclusions

We have described an accurate and efficient MM model, called EHMM, for matching GPS data to a digital map. The model comprehensively considers elements such as the shortest distance, heading direction, traffic regulation, topology and shape similarity. Compared with the existing HMM models, the obvious improvements of the EHMM model can be summarized as follows: (1) the EHMM model considers traffic rules and thus can ensure that vehicles obey traffic rules at post-matching points; (2) the EHMM model considers a shape similarity constraint, thereby ensuring that the probability distribution of the next state depends not only on the present state but also on past states. This strategy makes use of more information from historical trajectory points to reduce the matching error caused by the data uncertainty of the present trajectory point; and (3) the EHMM model considers topological information expressed in two different forms. When the sampling rate is high or moderate, explicit topological information is used in the EHMM model. When the sampling rate is low, implicit topological information is considered. Based on the ground truth data, the matching performance of EHMM was analyzed.

First, the matching accuracy of EHMM for a high sampling rate was evaluated. The results indicate that the matching accuracy of EHMM was higher than that of the reference models, namely, SM and HMM, for simple roads and complex roads. In addition, EHMM achieved better performance for simple roads than for complex roads; similar results were obtained for the reference models.

Then, the matching accuracy and running time of EHMM with respect to different sampling intervals were investigated. The results reveal that the matching quality of EHMM was stable (σ = 0.06) and less affected by the spatial location of vehicles than the reference models. With an increase in the sampling interval, the matching accuracy of EHMM significantly declined (*slope* = −0.74 × 10^−2^, *R*^2^ = 0.71); however, the running time yielded opposite results. The matching accuracy of EHMM was higher than that of the reference models for most of the sampling intervals. Prior to the sampling interval of 40 s, the running time of EHMM was shorter than that of the reference models. After that, the computation time of EHMM was long but substantially shorter than the sampling interval. Thus, the EHMM model was appropriate for operation with a low sampling rate.

The matching performance of EHMM for the actual trajectories of freight cars was verified. The results reveal that the matching accuracy of EHMM was significantly higher than that of the reference models on actual roads. The running time of EHMM was notably shorter than that of the reference models. The matching results of EHMM retained the topological adjacency between two roads and complied with traffic regulations better than the reference models. Moreover, the EHMM model is competitive relative to the other newer and more efficient models in terms of matching accuracy.

## Figures and Tables

**Table 1:** Evaluation of matching performance among MM models based on dataset 2.

Routes	Matching Accuracy			Running Time Per Point (ms)		
	EHMM	SM	HMM	EHMM	SM	HMM
Route 2	0.89	0.81	0.84	196	202	200
Route 3	0.90	0.76	0.80	98	138	134
